# The Psychometric Properties of the ACE-IQ Questionnaire’s Binary and Frequency Scoring Methods in a Chilean Community Sample

**DOI:** 10.3390/children12030340

**Published:** 2025-03-08

**Authors:** Maria-Pia Santelices, Maria-Carolina Velasco-Hodgson, Catterina Ferreccio, Catalina Undurraga, Karla Carvajal-Araneda

**Affiliations:** 1 School of Psychology, Pontificia Universidad Católica de Chile, Santiago 7820436, Chile; c.undurraga@uc.cl; 2 Early Adversity and Abuse Research Center, CUIDA, Santiago 7810000, Chile; mvelascoh@uc.cl; 3 School of Social Work, Pontificia Universidad Católica de Chile, Santiago 7820436, Chile; 4 School of Medicine, Pontificia Universidad Católica de Chile, Santiago 8320165, Chile; cferrecr@uc.cl; 5 School of Psychology, Universidad de Valparaíso, Valparaíso 2360102, Chile; karla.carvajal@uv.cl

**Keywords:** adverse childhood experiences (ACEs), health risk behaviors, ACE-IQ, Chile, validation, Marshall’s trauma scale

## Abstract

**Background/Objectives:** Adverse childhood experiences (ACEs) impact social, emotional, psychological, and physical development, often leading to health risk behaviors in adulthood. Instruments like the Adverse Childhood Experiences International Questionnaire (ACE-IQ) are essential for assessing ACEs globally and confirming their association with health outcomes in adulthood. **Methods:** This study evaluates the ACE-IQ’s validity in a Chilean cohort by analyzing the prevalence of ACEs and the instrument’s psychometric properties. Structural validity, internal consistency, and concurrent validity were assessed using the Marshall Scale as a comparative measure. Additionally, binary and frequency scoring methods were compared. **Results:** Structural validity analyses showed the best fit for three- and four-dimensional models using frequency scoring. The overall internal consistency of the scale was adequate (α > 0.7), although dimensions such as childhood neglect and violence outside the home demonstrated lower internal consistency. Concurrent validity showed significant positive correlations between ACE-IQ scores (both binary and frequency methods) and the Marshall Scale. **Conclusions:** The ACE-IQ demonstrates adequate reliability for the full scale, with strong evidence of construct validity using the frequency scoring method and concurrent validity for both scoring methods. These findings support the ACE-IQ’s use for measuring childhood adversities in Chile and assessing their association with adult health outcomes.

## 1. Introduction

### 1.1. The Impact of Adverse Childhood Experiences (ACEs) on Health

Over 20 years ago, Felitti et al. (1998) published a groundbreaking study on the long-term impact of adverse childhood experiences (ACEs) on health risk behaviors in adulthood [1]. Over time, this study has evolved from an epidemiological scientific article to being widely recognized as a critical comprehensive framework in public health [2]. Felitti et al. demonstrated that ACEs are much more prevalent than previously believed, using a sample of over 17,000 individuals from middle-class backgrounds [1]. Additionally, their research confirmed a robust relationship between adverse experiences during childhood and adult emotional health, physical health, and leading causes of mortality in the United States. Risks increased as ACEs increased in a dose–response relationship [1,3].

Subsequent studies have also established a relationship between these experiences and a higher prevalence of common health risk factors and long-term disease causes in adulthood, including cardiovascular diseases, diabetes, and cancer, among others [4]. Furthermore, there is a higher prevalence of subsequent psychopathology, with the number of ACEs serving as a predictor of mental illnesses such as depression, bipolar disorder, substance abuse, and post-traumatic stress disorder, along with other conditions [5].

The mechanism explaining this dose–response relationship involves brain-level changes. Childhood exposure to adversities can lead to toxic stress, resulting in disruptions in children’s neurodevelopment [6]. This manifests as dysregulation in the stress response, impairment in executive functioning, alterations in the endocrine and immune systems, genetic regulatory mechanisms, and other related effects. These alterations consequently lead to social, cognitive, and emotional dysfunction, an increase in risky behaviors, difficulty in forming healthy relationships, the early onset of diseases, and socio-emotional problems, and could ultimately result in premature death [4,7]. Therefore, childhood exposure to experiences such as abuse, neglect, domestic violence, and other stressors within the home significantly impacts individuals’ developmental trajectories, with consequences throughout the lifespan, that can even be passed down to future generations [8].

Most ACE studies have continued to use the ten adversities assessed by Felitti and colleagues in the original questionnaire (physical abuse, emotional abuse, physical neglect, emotional neglect, sexual abuse, household substance abuse, household incarceration, domestic violence, parental mental illness, and separation/divorce/loss of a parent) [1]. However, it has been noted that these 10 ACEs are not the only forms of adverse experiences that can occur in childhood, causing negative effects on developmental trajectories [9]. Therefore, to significantly improve the prediction of physical and mental health problems, it has been recommended to include other adverse experiences present at different levels of an individual’s ecology, such as peer violence and community violence [9,10]. Adverse childhood experiences have been defined by several prior studies and are well conceptualized by them [1,9,10,11].

### 1.2. The Development of the Adverse Childhood Experiences International Questionnaire (ACE-IQ)

In 2011, recognizing the significant interest in developing policies informed by the ACE framework, the World Health Organization (WHO) expanded the original ACE survey and developed the Adverse Childhood Experiences International Questionnaire [11]. This questionnaire covers, in addition to the original ten adverse experiences, a broader range of adverse events that individuals may encounter during childhood. The overarching objective behind developing the ACE-IQ was to provide an instrument capable of evaluating childhood adversities worldwide, studying their potential implications in different countries, and enabling international comparisons of ACEs’ prevalence and consequences, including collective violence [11]. The ACE-IQ assesses the occurrence of 13 categories of ACEs in adults, grouped into four dimensions. Each category of adversity occurrence is scored as 1, providing an overall score ranging from 0 to 13. The evaluated dimensions are as follows:

- Parents/caregivers’ physical and emotional neglect;
- Abuse, including physical and emotional abuse experienced at home and sexual abuse;
- Family dysfunction, encompassing one or more household members engaged in alcohol or drug abuse; a family member incarcerated; a household member with mental illness; a household member subjected to violence; or single or absent parents, parental separation, or divorce;
- Violence outside the home, including bullying, community violence (e.g., witnessing someone being assaulted/killed, fights), and collective violence (e.g., exposure to wars, terrorism, police, or gang fights).

The WHO has also encouraged the translation and inclusion of the ACE-IQ in comprehensive health surveys with the additional goal of testing the reliability and validity of this questionnaire in different countries [12]. This endeavor has been successfully accomplished; indeed, a recent systematic review reports that the instrument has been utilized in community samples from more than 29 countries across various income levels, including India, Turkey, Pakistan, China, Germany, the United States, Canada, the United Kingdom, Egypt, Kenya, Malawi, Sierra Leone, Tanzania, and Israel, revealing different prevalences of the sub-dimensions depending on the country [13]. This systematic review, encompassing 63 articles, concluded that on average, 75% of respondents in community samples experienced ACEs, with an average of three adversities per person. Emotional abuse and bullying had higher prevalence rates. Additionally, it was observed that in general, men experienced more ACEs but were under-represented in the samples [13].

Nonetheless, there is difficulty in the comparison of prevalences between countries because the WHO proposed two scoring methods to calculate ACE exposure (i.e binary and frequency scoring) and suggested comparing the results between both methods to ascertain “the most appropriate approach to determine an accurate overall ACE score for a participant” [14] (p. 1). Both scoring methods involve dichotomizing the 13 ACE categories into “non-exposure or 0” and “exposure or 1”, leading to a total score range from 0 to 13 [14]. However, these methods differ in their approach to quantifying exposure levels within these categories. The frequency scoring method considers the intensity or frequency of exposure and varies between the different kinds of ACEs. For instance, for sexual abuse, a single incident constitutes exposure. Conversely, for physical abuse, repetitive incidents are required to constitute exposure. However, the binary scoring method adopts a lower threshold for identifying ACE exposure. Under this method, any experience of adversity is regarded as exposure. For instance, being yelled at or sworn at even once is categorized as emotional abuse. However, challenges arise because most of the research that uses the instrument does not report or compare both coding systems. For example, none of the studies included in the most recent systematic review on the use of the ACE-IQ conducted a comparison between these two scoring systems [13]. Moreover, some studies did not even specify the scoring method they employed. Consequently, comparing prevalences becomes problematic. One recent study directly compared prevalences between the frequency and binary versions, revealing substantial differences in total ACE scores based on the chosen scoring method [15]. Also, Kidman et al. coded the instrument with both scoring methods, and they observed that frequency scoring yielded much lower total ACE scores but that the association with depressive symptoms was similar in magnitude to the binary version [16].

### 1.3. Psychometric Properties and International Validation of ACE-IQ

Regarding the instrument’s psychometric properties, numerous validation studies have been conducted in different countries [16,17,18,19,20]. Kazeem showed in a Nigerian sample that the instrument had good internal validity with a Cronbach’s alpha of 0.80 and showed that the ACE-IQ total score correlated with the CTQ [17]. In this study, six subscales were considered: marriage, relationship with parents/guardians, family environment, peer violence, witnessing community violence, and exposure to war/collective violence. Kidman et al. also showed that the ACE-IQ had good predictive validity by examining the association between the overall experience of adverse events and depression measured by the BDI score [16]. In a validation with a sample from Hong Kong, Ho et al. demonstrated that the instrument showed good content validity and internal validity with a Cronbach’s alpha of 0.80 [18]. They considered three subscales: “childhood maltreatment”, “family/household dysfunction”, and “violence outside the home” with Cronbach’s alphas of 0.74, 0.62, and 0.60, respectively [18]. In a French validation, Tarquino and colleagues considered the following dimensions that emerge from the structure of the original instrument: childhood maltreatment, family dysfunction, and violence outside the home, with Cronbach’s alphas ranging from 0.77 for child maltreatment to 0.62 for household disruption, and 0.41 for violence outside the home [20].

Regarding the structure of the instrument, Kidman et al. tested the instrument on a Malawian sample using principal component analysis [16]. They found a three-component solution comprising “household dysfunction”, “abuse”, and “neglect” components, but this solution did not include three of the thirteen ACEs. In a Mexican validation of the instrument, Téllez et al. conducted an exploratory factor analysis, finding five factors with adequate internal consistency: household violence, sexual abuse, family dysfunction, peer violence, and community violence with Cronbach’s alpha values for reliability of 0.86, 0.90, 0.72, 0.69, and 0.69 respectively [19].

Observing the differences in the categories reported in all the previous studies makes it clear that there is not a standard definition of the categories that comprise the ACE-IQ. This lack of clarity regarding its dimensions was also reported by Pace et al. after conducting a systematic review [13]. So, in this study, we will test the factor structure of the ACE-IQ with the aim of comparing the following solutions in a Chilean sample is to determine which one is the best fit: a three-factor solution (household dysfunction, childhood abuse, and external violence) or a four-factor solution (household dysfunction, childhood abuse, external violence, and childhood neglect). The rationale for the three-dimension model is based on a two-factor solution for the ten ACE questionnaire plus a third dimension of violence outside the home and is consistent with that reported by Tarquinio et al. [20]. On the other hand, the four-dimension solution is based on the structure reported by Pace et al. in their systematic review [13].

This study addresses the need to compare the psychometric properties of the ACE-IQ scale in both scoring systems and in three- or four-dimension models to offer guidance for its application. Furthermore, psychometric data for this instrument in Latin America are currently lacking, with just one study on psychometric properties that considered specific adaptations to Mexican culture [19]. Consequently, no information is available on the instrument’s psychometric properties for its use in the Chilean context. This research seeks to provide reliable information for using the ACE-IQ in Chile and other South American countries. The results will facilitate international comparisons and address the knowledge gap in ACE studies in Latin America, providing the necessary data to guide public policies that address early adversities and their consequences.

Given the context described above, the primary objective of this research was to validate the ACE-IQ scale in a Chilean community sample, analyzing the prevalence of adverse childhood experiences and the psychometric properties of the instrument in terms of (1) structural validity, through confirmatory factor analysis; (2) internal consistency, using Cronbach’s alpha and categorical omega as an index; and (3) concurrent validity, using the Marshall Scale as a comparison instrument. Additionally, this investigation entailed a comparative analysis of both scoring methodologies.

## 2. Materials and Methods

### 2.1. Design

This study had a cross-sectional design, was nested inside a prospective cohort study, and was approved by the Ethics Committee of Pontifical Catholic University of Chile. The studied sample was drawn from the “Maule Cohort (MAUCO) of chronic diseases in Chile 2014–2024” project of the Advanced Center for Chronic Disease [ACCDiS]. The MAUCO project protocol was approved by the Ethics Committee of the Pontifical Catholic University and by the Maule Regional Health Service [21].

### 2.2. Participants

Regarding the composition of the sample, it was a randomized sample stratified by sex, and it consisted of 705 participants enrolled in MAUCO before the year 2020, with the following inclusion criteria: (a) being 65 years old at enrollment in our study; (b) having complete questionnaires and medical examinations; and (c) having at least an elementary education and the ability to autonomously read the survey and record their answers in a tablet.

### 2.3. Procedure

Initially, a random sample was drawn from the individuals within the database of the “Maule Cohort (MAUCO) of chronic diseases in Chile 2014–2024” project. Subsequently, each selected person was contacted via telephone by a trained administrative assistant. During this call, they were invited to participate using a standardized script that thoroughly outlined all components of the informed consent process. Upon their agreement, an appointment was scheduled for them to visit the MAUCO module in Molina.

Upon arrival at the MAUCO module, participants were greeted by a psychologist from the research team. The psychologist provided a detailed explanation of the informed consent, distributed the survey for self-administration, and remained vigilant for any signs of physical or emotional distress among the respondents, prepared to activate appropriate support protocols if necessary.

In 2023, all participants from the Maule Cohort (MAUCO) were invited to a session where they received comprehensive feedback on the research findings, including those from the study presented in this article

### 2.4. Instruments

(a) Adverse experiences childhood: The Adverse Childhood Experiences International Questionnaire [ACE-IQ] was used: this is a retrospective self-report instrument for adults that investigates the occurrence of 13 categories of adverse experiences before the age of 18. The ACE-IQ was created by the World Health Organization [WHO] [11]. The experiences included in the questionnaire are as follows: emotional neglect, physical neglect, emotional abuse, physical abuse, sexual assault, caregiver used drugs/alcohol, caregiver presented psychopathology, caregiver deprived of liberty, parents separated or divorced or death of caregiver, witness of domestic violence, bullying/aggression by peers, witness of community violence, and exposure to political, collective violence or war. Exposure to each specific category of childhood adversity was coded as a binary ACE score (presence or absence of that type of experience) such that participants’ total ACE scores indicate how many types of adversities they were exposed to (0 to 13 ACEs). In the application of the instrument, two questions were added to differentiate the type of exposure to collective violence. Specifically, violence caused by the military and police was differentiated from that caused by terrorism, militias, or gangs, given that political violence and public order forces have marked the history of Chile; this type of adjustment to the instrument has also been carried out in other studies, such as in Saudi Arabia, China, and Korea, and these adjustments are promoted by the WHO to adjust the instrument to the realities of each country [22,23,24]. The instrument was scored using both methods to calculate ACE exposure (binary and frequency). Both scoring methods involve dichotomizing the 13 ACE categories into “non-exposure or 0” and “exposure or 1”, leading to a total score range from 0 to 13. The frequency scoring method considers the intensity or frequency of exposure and varies between the various kinds of ACEs [14]. However, the binary scoring method adopts a lower threshold for identifying ACE exposure. Under this method, any experience of adversity is regarded as exposure [14].
(b) Marshall Trauma Scale: The Marshall Scale is a questionnaire that determines the memory of the occurrence of maltreatment during childhood [25]. This scale is a brief instrument, whose external validity has been confirmed in Chile, obtaining a Pearson correlation coefficient of 0.88 [26]. The scale evaluates the presence or absence of early adverse experiences through seven items: (1) traumatic separation from the father, mother, or caregiver for more than one month; (2) experience of having suffered significant physical punishment; (3) being left with physical damage after having been punished; (4) having witnessed physical violence between parents or caregivers; (5) alcohol or drug abuse by a family member; (6) forced sexual contact by a relative; and (7) forced sexual contact with a non-family member. This scale was recently used in Chile for the initial validation of the Brief Childhood Trauma Questionnaire [CTQ-SF] [27].

It is well known that scales with the purpose of measuring retrospective information have been questioned in the past due to the possible effect of memory bias, and this was raised by Pace et al. [13] as one of the possible explanations for the difference in scores in some adversities between children and adults, with Felitti et al. declaring that memory bias in these questionnaires could result in the underestimation of the presence of such experiences [1]. According to other authors [28,29], there are differences between ordinary memories and traumatic memories, with the latter having the power to become autobiographical memories, which become present in a recurrent manner and, despite being fragmented, can be evoked with strategies that promote their recollection [30]. For this reason, this study decided to include the ACE-IQ, where at least two questions are presented for most of the ACEs, to increase the probability of evoking the memory of each adversity.

### 2.5. The Translation of the ACE-IQ

The questionnaire is freely available and was translated and adapted for the Chilean study following World Health Organization guidelines. Given the above, the first step consisted of the translation of the instrument into Spanish by two psychologists with professional competence in English and in the subject that is addressed in the questionnaire. In the second step, the translation was reviewed by a group of experts in the area. The third step was a back-translation process developed by two bilingual members of the Translation Review Committee of the National Child Traumatic Stress Network (NCTSN, U.S.) to review the adequacy of the translation of the original instrument into Spanish. The fourth step of the process was reviewing the translation with a professional team working on interviews and home visits to adjust the instrument’s language to the population to which it is directed. The fifth step was a pilot application of the survey to 12 people, with similar sociodemographic characteristics to the study sample, which allowed for an evaluation of the implementation of the instrument. This pilot application was carried out to test the instrument’s application digitally and explore the possible emotional impact that people who answered the questionnaires could experience. Through this translation process, the understanding of the items was checked and corrected, and during content review and piloting, the Action Protocol was adjusted based on the possible emotional impact on participants due to the application of the questionnaire.

### 2.6. Statistical Analysis

For data analysis, 54 participants of the initial sample of 705 were excluded due to missing information in any of the ACE-IQ questions. Thus, the analysis was carried out on a sample of 651 participants with complete information. To evaluate potential biases due to sample reduction, the characteristics of both subsamples were analyzed (that is, with and without missing information in the ACE-IQ), and no significant differences were found between them in the variables analyzed (age, sex, education, and marital status), as shown in Table 1.

To examine the structural validity of the ACE-IQ, a confirmatory factor analysis (CFA) was conducted. This analysis allows for the assessment of goodness of fit for the structure of a questionnaire developed or found in the previous literature. In this case, models of three and four factors were tested. Simultaneously, these models were tested for binary and frequency corrections. The factor models were specified using a polychoric matrix for each model, as this is the recommended procedure when analyzing categorical variables [31,32]. The estimator used for these purposes is WLMSV, which does not require a distributional assumption check [33]. To assess model fit, fit indices including the comparative fit index (CFI), the Tucker–Lewis index (TLI), the root mean square error of approximation (RMSEA), and the standardized root mean squared residual (SRMR) were analyzed [34]. A good model is one with an SRMR close to or less than 0.08, RMSEA close to or less than 0.06, and CFI and TLI close to or greater than 0.95 [33].

Cronbach’s alpha and categorical omega indices were used to estimate internal consistency. Cronbach’s alpha allows for a quantification of the consistency of the variation in the items of a scale [35]. On the other hand, the categorical omega index is a robust estimate of internal consistency, because it admits the non-fulfillment of assumptions (the equality of factor loadings and that the variables are continuous, in the case of Cronbach’s alpha) [36]. In addition, it uses polychoric correlations at the base, which, as mentioned above, are more appropriate when analyzing dichotomous variables.

While categorical omega is a more appropriate indicator of internal consistency in this case, Cronbach’s alpha was still calculated. This was carried out to compare the results with those of other, previously conducted studies of the psychometric properties of the ACE-IQ, in which Cronbach’s alpha was used to estimate the internal consistency of the scale.

Finally, a concurrent validity analysis was conducted with the Marshall Trauma Scale (MTS), using Spearman correlation analysis and simple linear regression. According to Cohen, effect sizes (correlation coefficients) are described as small (0.10), medium (0.30), and large (0.50) [37]. In this case, it is hypothesized that the correlation between ACE-IQ and MTS scores will be significant (*p* < 0.05), be positive, and have a large effect size.

For simple linear regression, bootstrapping was performed, which is a method that allows for the confidence intervals to be estimated from the sample and for the quality of the estimate when assumptions are not met to be assessed [38].

The analyses were conducted using RStudio 2022.07.2 Build 576 and the packages “psych” Version 2.3.12, “corrplot” Version 0.92, “lavaan” Version 0.6-17, “semTools” Version 0.4-14, “MASS” Version 7.3-60 and “boot” Version 1.3-28.1 [39,40,41,42,43,44].

## 3. Results

### 3.1. Descriptive Analysis

Among the participants, 54% were women, the mean age was 48 years, most lived with a partner, and most had a high school education. Table 2 shows the main characteristics of the participants, and Table 3 indicates ACE prevalence in the ACE-IQ binary and frequency scores.

### 3.2. Factorial Analysis

In total, four factor models were estimated: two for three dimensions with binary and frequency scorings, and two for four dimensions with both scoring methods. For the three-factor and four-factor models, confirmatory factor models were estimated by clustering as presented in Table 4. The (robust) fit indices show that only the frequency scored models (M2 and M4) fit adequately.

Regarding the factor loadings of the model, most are close to 0.50, which is considered good. Despite this, there are some factor loadings considered reasonable to poor < 0.4 [30] as illustrated in Table 5.

### 3.3. Internal Consistency

The internal consistency of the overall scale is adequate (>0.7). However, the dimensions of childhood negligence and violence outside the home show lower internal consistency (Table 5). This may be due to the number of items in each category. Both coefficients (categorical omega and Cronbach’s alpha) are known to be biased by the number of items (both require the number of items for their computation). For example, if the domain of *violence outside the home* were to maintain average correlation between its items but see an increase in the number of items within it, its internal consistency would increase considerably. It is also important to note that internal consistency is also affected by how the participant perceives the content of each item [45], as evidenced by Table 6

### 3.4. Concurrent Validity

To conduct the concurrent validity analysis, the magnitude of the relationship between the total score of the ACE-IQ (binary and frequency scoring) and the Marshall Trauma Scale (MTS) was first evaluated. Spearman correlations were used for this purpose. The results indicated that the ACE-IQ score (binary and frequency scoring methods) correlated significantly and positively with the MTS (ρ(651) = 0.73, *p* < 0.001; and ρ(651) = 0.75, *p* < 0.001; respectively). Table 7 shows the results of the regressions between the ACE-IQ (binary and frequency scoring) and the MTS.

Therefore, both models are adequate (when using binary or frequency scoring methods for the ACE-IQ) and the best model is the second one (frequency scoring method). In this model, for each point obtained in the ACE-IQ, the MTS score increases significantly by 0.47 points. *R*^2^ indicates that the ACE-IQ explains 60% of the variance in the level of trauma measured with the MTS.

It is important to mention that each estimated model obtained a significant fit to the data (significant ANOVA F-value) and that, due to the non-fulfillment of the normality assumption, confidence intervals for the intercept and predictor were estimated by bootstrapping (resampling performed 1000 times). The estimates of the bootstrapped confidence intervals were the same as the estimates made by the regressions, so it can be confirmed that the results are adequate.

## 4. Discussion

Our study is the first in Chile to examine the psychometric properties of the ACE-IQ in South America, and it intends to provide a measure for future studies in Chile. This country has a high prevalence of ACEs; a national survey shows that around 40% of the urban population has experienced four or more ACEs in their lifetime [46]. In the past, Chilean studies included a fraction of ACEs in their studies; in the present, the availability of an instrument like the ACE-IQ with a wider range of ACEs provides the opportunity to assess a more realistic prevalence of ACEs in the Chilean context. Given the association of ACEs with later life consequences, it is crucial to have a measure for use in future studies, which need to explore this association in Chile.

This study embraced the ACE-IQ developers’ advocacy of the utilization of both scoring methods in research settings to enable cross-study comparisons; the present study does not intend to make a comparison between different cultures since this comparison was already made by Pace et al. [13], which detail the limitations of making such a comparison because most studies using the ACE-IQ only include one method of scoring (binary or frequency) and some studies lack clarification regarding the correction method used to score the questionnaire, which makes it even more difficult to compare results at a cross-cultural level. To address this challenge, all studies using the ACE-IQ should report on the scoring system employed or, ideally, compare both scoring methods within their samples.

The results of this study suggest that the ACE-IQ, translated into Spanish and adapted to the Chilean context, has appropriate full-scale reliability (using binary and frequency scorings), with evidence of adequate construct validity (for frequency scoring) and concurrent validity (using both methods of scoring).

This is in line with other studies [20,47]. However, it is important to note that in studies such as the Kidman and Swinge studies, analyses were performed using the score for the totality of the items, obtaining a comparatively higher estimate of internal consistency [16,47]. For this reason, the coefficients obtained in the present study are more similar to those in Tarquinio et al., in which the score was calculated for 13 categories, considering the corresponding correction methods [20]. In addition, the dimensions were grouped according to WHO (for three domains) and Pace et al. (for four domains) guidelines [13,48]. In this sense, as can be observed in the present study, the internal consistencies for the domains household dysfunction, external violence, and child neglect are rather low and inadequate. This same scenario is presented in the Tarquinio paper (for three dimensions) [20].

There are some reasons why this result might have occurred, including the following:

- The number of items affects the estimates of internal consistency [49]. In the Kidman and Swingen works, by using the scores of all items, without employing any prior method of scoring, the number of items per domain increases, which could affect and improve the estimates of internal consistency coefficients [16,47].
- The context can influence the perception of some items and, therefore, the scores obtained on these items [45]. In this case, as can be seen in Table 3, there are two items with factor loadings below 0.4. One corresponds to the category one or no parents, parental separation, or divorce, and the other to the category collective violence. In the first category, which belongs to the domain dysfunction in the home, to obtain the score, we included questions such as the following: “Were your parents ever separated or divorced? Did your mother, father or guardian die? Were you separated from your parents for a long period of time?” These questions could be perceived differently from each other, which causes a decrease in the consistency of the answers, affecting the total internal consistency of the domain.

On the other hand, the second item with low factor loading corresponds to the category collective violence, which belongs to the domain of external violence. In this case, as noted by Tarquinio et al., except for the fact that the categories bullying, community violence and collective violence are related to psychosocial violence, there is no real coherence between these categories [20]. Using the low factor loading criterion, if the collective violence category is eliminated, and only the internal consistency between the bullying and community violence categories is observed, the categorical omega value increases to 0.49 and 0.48 (binary and frequency correction, respectively).

For the reasons mentioned above, it is important in the future to review the validity of the instrument in detail. This could involve studying the instrument in other contexts and/or using the full item scores when assessing its psychometric properties.

One limitation of this study is the age range of the sample (37–66 years, M = 48), as many validation studies of the ACE-IQ have focused on younger populations. However, this characteristic also represents a strength given the increasing importance of population aging. ACEs have long-term consequences that extend into adulthood and older age, influencing mental health, physical health, and overall well-being. As global life expectancy rises, understanding how ACEs affect middle-aged and older adults is crucial for developing interventions and public health policies tailored to aging populations [50,51]. Therefore, while the age range may limit comparability with studies on younger samples, it also contributes valuable insights into the lasting impact of ACEs across the lifespan, addressing a critical gap in research on older populations. Future studies should incorporate younger populations and assess the psychometric properties within those groups.

Another limitation is the differences observed in the factorial structure and internal consistency of the scale. As future lines of research, it would be pertinent to review and improve those items that present difficulties in their interpretation, as well as to evaluate the psychometric properties of the ACE-IQ using the complete questionnaire with its total score. Likewise, the possibility of assigning a differential weight to each of the adverse experiences could be explored, recognizing that their impact is not uniform and that some may have greater relevance in the severity of trauma. These approaches would allow a more accurate and representative measurement of childhood adversity in different contexts.

Nevertheless, the initial validity evidence obtained is adequate, and it is expected that the use of the ACE-IQ will be an important contribution to the study of adverse childhood experiences in the Chilean population.

## Figures and Tables

**Table 1:** A Comparison of sociodemographic characteristics and variables of interest with and without missing values in the ACE-IQ in the sample of participants from the Maule Cohort (MAUCO) (N = 705).

Sociodemographic Characteristics and Variables of Interest	ACE–IQ Complete (*N* = 651)	ACE-IQ with Missing Values (*N* = 54)	*p* Value
Age (mean (SD))	48.41 (*SD* = 6.82)	48.74 (*SD* = 6.34)	0.71
Sex female	348 (53.5%)	33 (61.1%)	0.35
Partner status			
Single	104 (16.0%)	7 (13.0%)	0.70
Married or coupled	461 (70.8%)	39 (72.2%)	0.95
Separated or divorced	75 (11.5%)	6 (11.1%)	1.00
Widowed	11 (1.7%)	2 (3.7%)	0.60
Educational level			
Elementary education or less	134 (20.6%)	15 (27.8%)	0.28
Highschool education	417 (64.1%)	33 (61.1%)	0.78
College or postgraduate education	100 (15.4%)	6 (11.1%)	0.52

Note: *p* value for significant differences by group ≤0.05.

**Table 2:** Sociodemographic characteristics and ACE scores by sex in 651 MAUCO participants.

Characteristics	Women (N = 348)	Men (N = 303)	Total (N = 651)
Age (mean (SD)) (minimum–maximum)	48.1 (SD = 6.8) (37–66)	48.8 (SD = 6.8) (37–64)	48.4 (SD = 6.8) (37–66)
Partner status (%)			
Single	17.5	14.2	16.0
Married or coupled	67.8	74.3	70.8
Separated or divorced	11.8	11.2	11.5
Widowed	2.9	0.3	1.7
Educational level (%)			
Elementary education or less	19.0	22.4	20.6
High school education	66.4	61.4	64.1
College or postgraduate education	14.7	16.2	15.4
ACEs (Mean (SD)) *			
Binary scoring	5.7 (2.9)	5.1 (2.9)	5.4 (2.9)
Frequency scoring	3.4 (2.8)	2.5 (2.4)	3.0 (2.7)

Note. * *p* value for significant differences by group ≤0.05.

**Table 3:** ACE prevalence in ACE-IQ binary and frequency scoring by sex in 651 MAUCO participants.

Adverse Childhood Experience	Binary Scoring (%)	Frequency Scoring (%)
Emotional neglect	59.1	28.7
Physical neglect	34.9	16.3
Emotional abuse	54.5	19.0
Physical abuse	63.0	15.5
Sexual abuse	26.1	26.1
Substance abuser in household	34.9	34.9
Someone with mental health issues in household	10.9	10.9
Incarcerated household member	7.1	7.1
Caregiver dead, abandoned or separated	32.1	32.1
Domestic violence	67.3	43.8
Bullied	48.7	14.6
Community violence	71.9	15.7
Collective violence	31.2	31.2

**Table 4:** Confirmatory factor analysis fit indices for the ACE-IQ.

Model (Scoring Method)	CFI	TLI	RMSEA	SRMR	*χ* ^2^	*df*	*p*
M1 3F (binary)	0.86	0.83	0.10	0.07	92.23	62	0.008
M2 3F (frequency)	0.98	0.97	0.05	0.05	47.45	62	0.914
M3 4F (binary)	0.87	0.83	0.10	0.06	82.36	59	0.000
M4 4F (frequency)	0.98	0.97	0.05	0.05	45.48	59	0.902

Note. *n* = 651; CFI = comparative fit index; TLI = Tucker-Lewis index; RMSEA = root mean square error of approximation; SRMR = standardized root mean squared residual; *χ^2^* = chi-square goodness of fit test; *df* = degree of freedom.

**Table 5:** Factor loadings of ACE-IQ for three dimensions and four dimensions.

ACEs	Three Dimensions						Four Dimensions							
	Binary Scoring			Frequency Scoring			Binary Scoring				Frequency Scoring			
	HD	CA	EV	HD	CA	EV	CN	HD	CA	EV	CN	HD	CA	EV
ACE1		0.54			0.69		0.59				0.72			
ACE2		0.51			0.50		0.57				0.52			
ACE3	0.68			0.71				0.68				0.71		
ACE4	0.52			0.51				0.52				0.51		
ACE5	0.70			0.68				0.69				0.68		
ACE6	0.34			0.39				0.34				0.39		
ACE7	0.91			0.87				0.91				0.87		
ACE8		0.88			1.00				0.92				1.01	
ACE9		0.72			0.87				0.74				0.87	
ACE10		0.51			0.53				0.52				0.53	
ACE11			0.65			0.76				0.65				0.76
ACE12			0.76			0.74				0.76				0.74
ACE13			0.36			0.32				0.36				0.32

Note. HD = household dysfunction; CA = childhood abuse; EV = external violence; CN = childhood negligence. ACE1 = emotional neglect; ACE2 = physical neglect; ACE3 = alcohol and/or drug abuser in household; ACE4 = someone chronically depressed, mentally ill, institutionalized, or suicidal; ACE5 = incarcerated household member; ACE6 = one or no parents, parental separation, or divorce; ACE7 = household member treated violently; ACE8 = emotional abuse; ACE9 = physical abuse; ACE10 = contact sexual abuse; ACE11 = bullying; ACE12 = community violence; ACE13 = collective violence.

**Table 6:** Internal consistency of ACE-IQ (three dimensions and four dimensions).

	Three Dimensions				Four Dimensions			
	Binary Scoring		Frequency Scoring		Binary Scoring		Frequency Scoring	
Category	Categorical ω	Cronbach’s α	Categorical ω	Cronbach’s α	Categorical ω	Cronbach’s α	Categorical ω	Cronbach’s α
ACE-IQ	0.76	0.74	0.78	0.76	0.76	0.74	0.78	0.76
HD	0.58	0.51	0.61	0.54	0.58	0.51	0.61	0.54
CA	0.64	0.61	0.72	0.67	0.64	0.57	0.74	0.64
EV	0.46	0.44	0.41	0.43	0.46	0.44	0.41	0.43
CN	-	-	-	-	0.35	0.34	0.38	0.34

Note. HD = household dysfunction; CA = childhood abuse; EV = external violence; CN = childhood negligence.

**Table 7:** Simple regression of effect of ACE-IQ on MTS.

Scoring	Predictors	Marshall Trauma Scale		
		β	CI	*p*
Binary	Intercept	−0.74	[−0.90–−0.60]	<0.001
	ACE-IQ	0.40	[0.37–0.44]	<0.001
	*R*^2^/*R*^2^ adjusted	0.51/0.51		
Frequency	Intercept	0.07	[−0.03–0.16]	0.266
	ACE-IQ	0.47	[0.43–0.50]	<0.001
	*R*^2^/*R*^2^ adjusted	0.60/0.60		

Note. β = regression coefficient. CI = confidence interval.

## Data Availability

The data that support the findings of this study are available from the corresponding author upon reasonable request due to ethical reasons.
